# Analysis of Magnetic Resonance Image Signal Fluctuations Acquired During MR-Guided Radiotherapy

**DOI:** 10.7759/cureus.2385

**Published:** 2018-03-28

**Authors:** Adrian L Breto, Kyle R Padgett, John C Ford, Deukwoo Kwon, Channing Chang, Martin Fuss, Radka Stoyanova, Eric A Mellon

**Affiliations:** 1 Department of Radiation Oncology, Sylvester Comprehensive Cancer Center, Miller School of Medicine, University of Miami, Miami, USA; 2 Biostatistics and Bioinformatics Shared Resource, Sylvester Comprehensive Cancer Center, Miller School of Medicine, University of Miami, Miami, USA; 3 Miller School of Medicine, University of Miami Medical School, Miami, USA; 4 Clinical Cooperative Think Tank, Viewray Inc.

**Keywords:** glioblastoma, mri, principal component analysis

## Abstract

Magnetic resonance-guided radiotherapy (MRgRT) is a new and evolving treatment modality that allows unprecedented visualization of the tumor and surrounding anatomy. MRgRT includes daily 3D magnetic resonance imaging (MRI) for setup and rapidly repeated near real-time MRI scans during treatment for target tracking. One of the more exciting potential benefits of MRgRT is the ability to analyze serial MRIs to monitor treatment response or predict outcomes. A typical radiation treatment (RT) over the span of 10-15 minutes on the MRIdian system (ViewRay, Cleveland, OH) yields thousands of “cine” images, each acquired in 250 ms. This unique data allows for a glimpse in image intensity changes during RT delivery. In this report, we analyze cine images from a single fraction RT of a glioblastoma patient on the ViewRay platform in order to characterize the dynamic signal changes occurring during RT therapy. The individual frames in the cines were saved into DICOM format and read into an MIM image analysis platform (MIM Software, Cleveland, OH) as a time series. The three possible states of the three Cobalt-60 radiation sources—OFF, READY, and ON—were also recorded. An in-house Java plugin for MIM was created in order to perform principal component analysis (PCA) on each of the datasets. The analysis resulted in first PC, related to monotonous signal increase over the course of the treatment fraction. We found several distortion patterns in the data that we postulate result from the perturbation of the magnetic field due to the moving metal parts in the platform while treatment was being administered. The largest variations were detected when all Cobalt-60 sources were OFF. During this phase of the treatment, the gantry and multi-leaf collimators (MLCs) are moving. Conversely, when all Cobalt-60 sources were in the ON position, the image signal fluctuations were minimal, relating to very little mechanical motion. At this phase, the gantry, the MLCs, and sources are fixed in their positions. These findings were confirmed in a study with the daily quality assurance (QA) phantom. While the identified variations were not related to physiological processes, our findings confirm the sensitivity of the developed approach to identify very small fluctuations. Relating these variations to the physical changes that occur during treatment shows the methodical ability of the technique to uncover their underlying sources.

## Introduction

The use of magnetic resonance imaging (MRI) is rapidly gaining momentum in the management of cancer because of its improved diagnostic potential and wide availability. The non-invasive nature of MRI allows for frequent scanning and follow-up of patients. However, real-time imaging of patients during radiotherapy was not available until the recent release of the Tri-Cobalt-60 MRI-Guided RT (MRgRT) radiotherapy system MRIdian (ViewRay, Cleveland, OH) [1-3].

A typical treatment fraction on MRIdian yields thousands of images over the span of 10 to 15 minutes. Because of the short time of acquisition (250 ms per image) and the low-strength magnetic field (0.35T), these images are of low signal to noise ratio. However, this unique data allows for a glimpse in image intensity changes during RT delivery.

The aim of this study is to identify and quantify the MR signal variations during MRIdian treatment of a patient with glioblastoma. We use principal component analysis (PCA) to take advantage of the collective power of multiple images which were acquired during a single fraction. The identified patterns were confirmed in another subsequent fraction on the same patient and in a phantom study.

## Case presentation

The MRIdian platform consists of a rotating circular gantry with three Cobalt-60 sources spaced in 120 degree intervals from each other. All treatments utilize a step-and-shoot intensity modulated radiation therapy (IMRT) delivery technique and the platform has a distinct procedure for delivering radiation. First, the gantry will rotate into the delivery position required, with all sources locked in the OFF position during rotation. When the gantry is secured, the multi-leaf collimators (MLC) will open to provide the correct aperture for RT delivery and the sources move into the READY position. Once all of the MLCs are in position, the sources then traverse to the ON position and radiation is delivered to the patient. Whenever an alteration is required to the shape of the beam at any of the sources, the source will return to the READY state until its MLC assumes the new aperture required, whereupon the source will return to ON. In the MRIdian software, these states are visualized on the cine as follows: green indicating OFF, yellow indicating READY, and orange indicating ON.

The analysis was performed on a cine generated from the real-time tracking of RT treatment of a patient with glioblastoma. The patient was treated with 30 fractions at 60 Gy over six weeks and immobilized with a thermoplastic mask and surface coils surrounding the mask. A patient with a brain tumor was chosen for the study because of the essentially immobile head during RT and the reproducibility of the setup. Over 3000 MRIs are acquired over one treatment fraction (250 ms per image). The images are displayed in MRIdian’s console and the resulting movie captured in Ogg Vorbis (.ogv) format.

A script was created in MATLAB (MathWorks, Natick, MA) for converting the cine from .ogv file to pseudo-DICOM images. The individual frames of the video were extracted and rendered into greyscale. To speed the subsequent computation time, the cine were rescaled down from a 914 × 914 video resolution to 236 × 236 pixels. Synthetic DICOM headers were appended to the image arrays and saved as a dynamic series.

**Figure 1:**
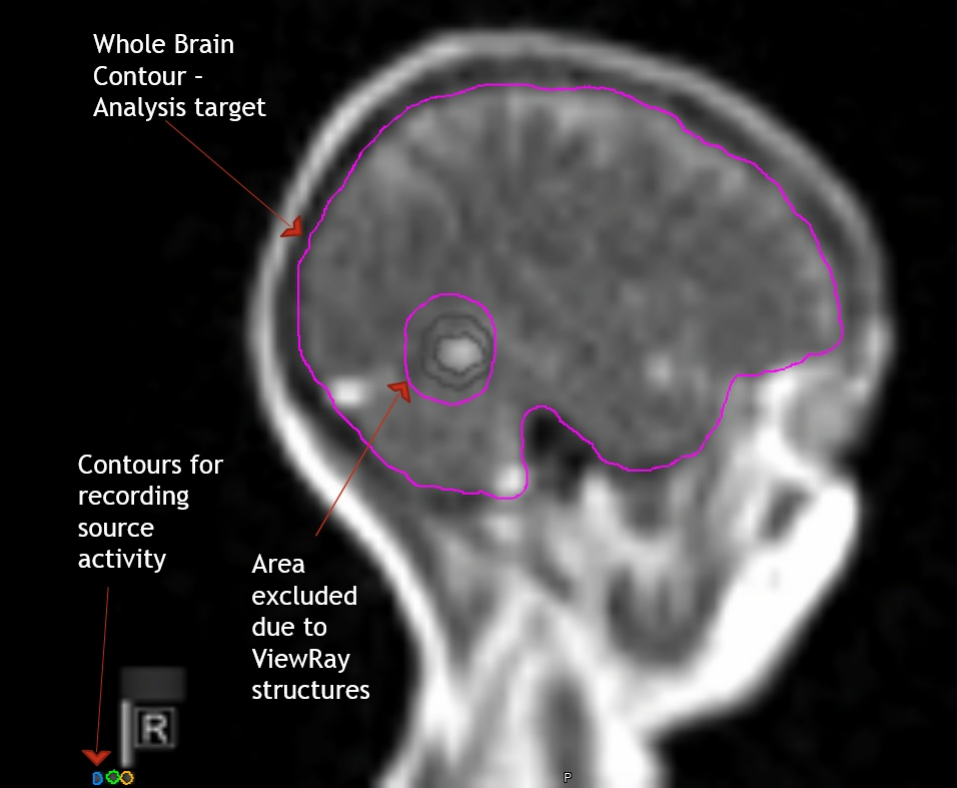
Example of cine content and segmentation. A single frame of cine from a patient with glioblastoma, displayed in MIM (MIM., Cleveland, OH). The high intensity signal in the posterior corresponds to the fluid in the surgical resection cavity. The contours in grey around the lesion are the tracking (inner) and boundary (outer) contours. Both contours are built into the cine and cannot be removed. The Volume of Interest (VOI), the brain, is contoured in pink, avoiding the areas of the tracking and boundary contours. The analyzed data matrix contains in its rows the cine intensity changes in time for each voxel in the VOI. Three boxes (Regions of Interest (ROIs)) are contoured over the Cobalt-60 indicators.

**Figure 2:**
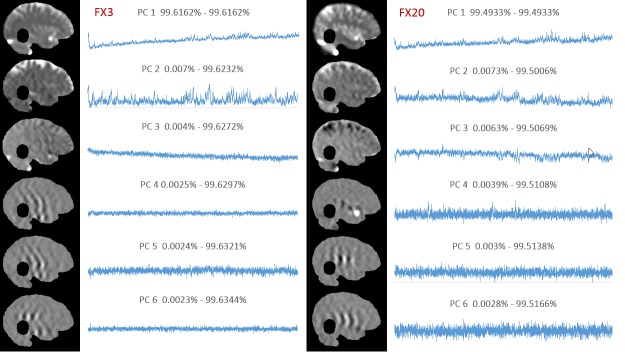
Principal component analysis of treatment fractions 3 and 20. Principal component analysis on Fraction 3 (left) and Fraction 20 (right). For each fraction, the principal components (PCs) are represented as a function of time; their corresponding weights are visualized as a 2D map on the brain mask. The numbers above the PCs indicate the percentage of total variance explained by the corresponding PC.

The DICOM data were imported into the imaging analysis platform MIM (MIM Software, Cleveland, Ohio) and read as a single time series. The patient brain was contoured (Volume of Interest, VOI), excluding the MRIdian tracking and boundary structures (Figure 1). In addition, a Region of Interest (ROI) was drawn around each of the indicators for the Cobalt-60 sources. A custom Java-based plugin for MIM was created to perform PCA. PCA is a standard statistical technique for decomposition of large multivariable datasets along the axes of largest variations [4]. PCA is applied to the data matrix, containing in its rows the cine intensity changes in time for each voxel in the VOI. The Java-PCA routine visualizes the shape of the principal components (PCs) as a function of time, together with their weights in each voxel, displayed as a spatial map (Figure 2). The intensity of Cobalt-60 source indicators was extracted, using MIM utilities.

**Figure 3:**
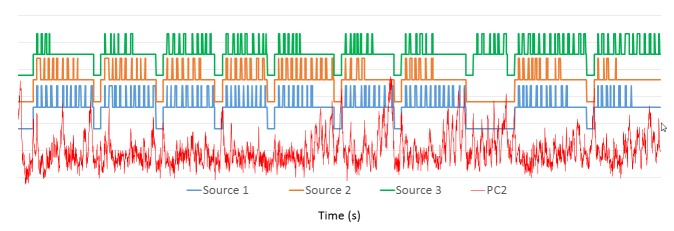
Signal of 2nd principal component with source activity. Cobalt-60 sources co-registered in time with PC2 from Fraction 3. The changes in the Cobalt-60 activity correspond to: OFF – Bottom state, READY – Middle state, ON – Upper state. Each of the three sources is offset for legibility.

**Figure 4:**
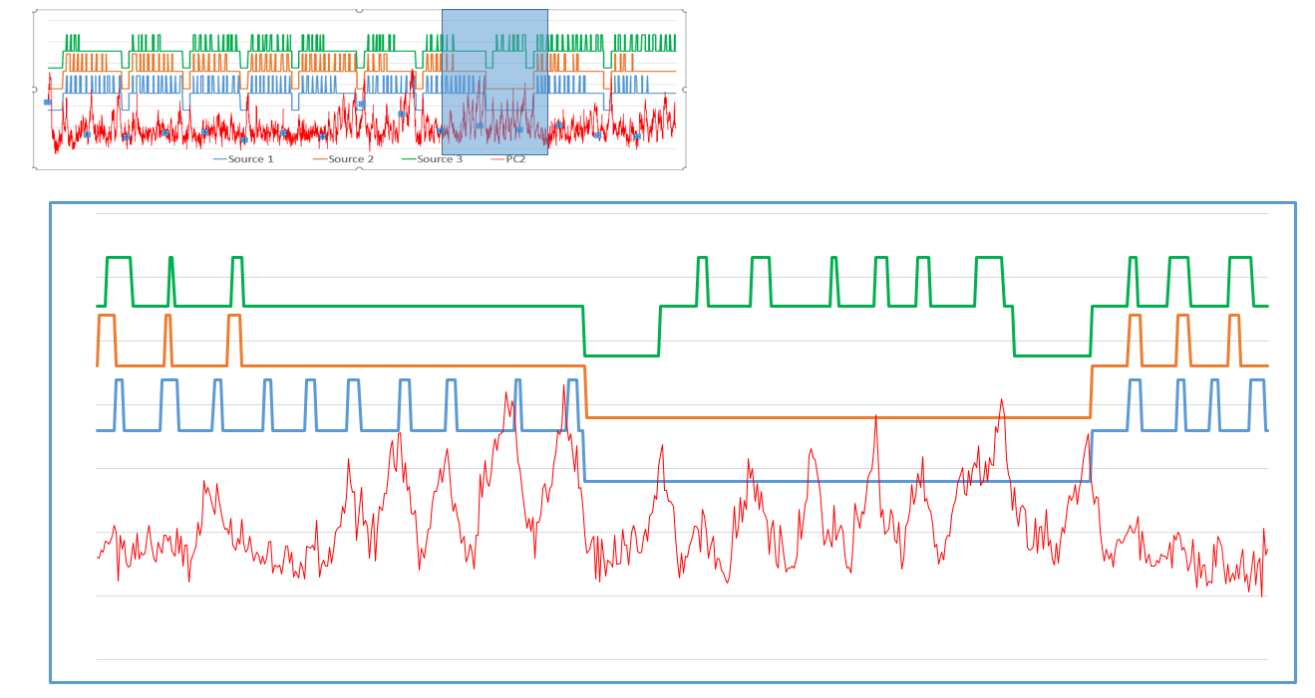
Detail of fraction 3 source and signal activity. Enlarged view of the segment in the box from Figure 2. Larger signal variations in PC2 (red) are present when a single source is active compared to when two or more of the sources are in operation.

**Figure 5:**
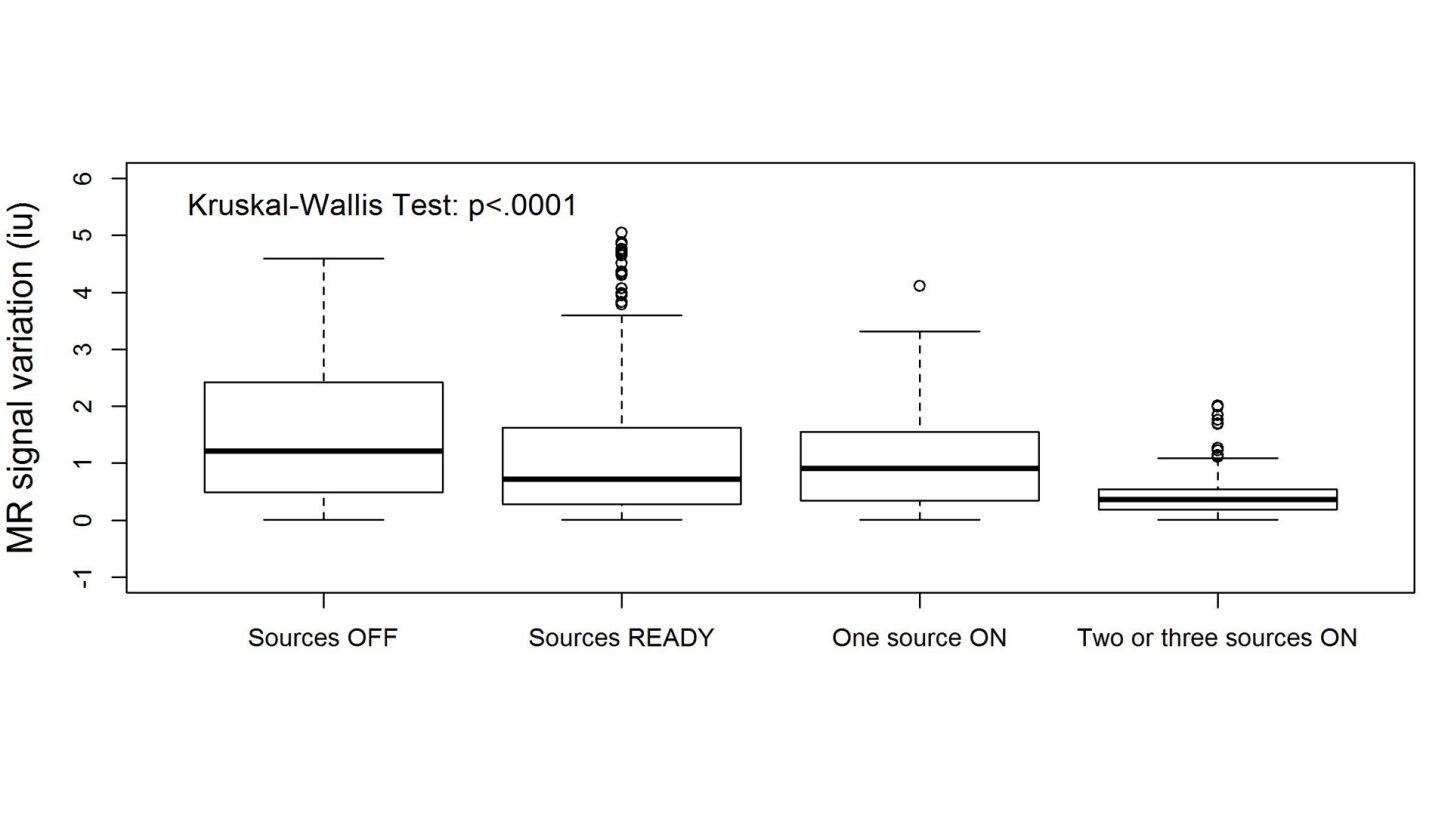
Statistical analysis for 3rd and 20th fractions performed on patient. Box plots of signal intensities in PC2 as a function of Cobalt-60 states. The signal variation decreases significantly as more sources are in operation (Kruskal-Wallis test, p < 0.0001).

We applied the procedure to the 3rd and 20th treatment fractions performed on the patient (3183 and 3491 images, respectively). The results of the PCA analysis are shown in Figure 2. Note the large similarity between the shapes of the PCs and the distribution of their weights. The high-intensity areas along the surface of the brain contour coincide with known cerebrospinal fluid (CSF) spaces. As these are higher intensity signals, their dynamic changes are more readily reflected in the weight projection mask. There is a gradual upward trend in the first PC, representing a monotonic change in overall signal intensity that may be related to subtle magnetic field drift, small changes in the relative position of the patient to RF coil, receiver gain drift or other system electronics-related changes over time. The second PC is more interesting: it captures significant changes in the image intensity. In order to uncover the source of these variations, the Cobalt-60 indicators and the second PC were co-registered in time (Figure 3). A clear pattern in PC2 emerged from this comparison. We found that the PC2 values were highly variable at periods where all or some of the Cobalt-60 sources were OFF as opposed to the ON-position. These variations were also apparent in the case of one Cobalt-60 source ON and two Cobalt-60 sources OFF (Figure 4). To quantify this observation, we removed noise from the signal with a wavelet denoising method using the Daubechies least asymmetric wavelet with 4 vanishing moments up to level 4 [5]. We performed a Kruskal-Wallis test on the denoised signal to examine whether there are statistically significant differences among groups. We presented the PC variations in a boxplot relative to four positions of Cobalt-60 sources (Figure 5). It is clear that the largest variations are related to the OFF position all Cobalt-60 sources. During this phase of the treatment, the largest metallic components, the gantry and the MLCs, are moving. Conversely, when all Cobalt-60 sources were ON, the image signal fluctuations were minimal, related to very little mechanical motion. At this phase, the gantry, the MLCs, and sources are fixed in their positions. When only one or two sources are ON, the other(s) are either in the READY or OFF position, which involves shuttling of the Cobalt-60 sources and adjustment or closure of the MLCs, producing fluctuations within the image.

**Figure 6:**
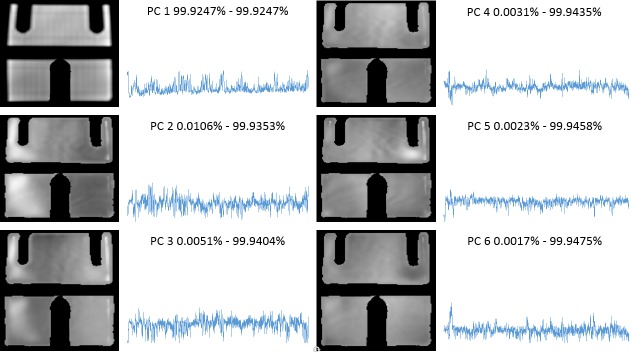
Principal component analysis of fraction 3 on imaging phantom. Principal component (PC) analysis on cine from a daily quality assurance (QA) phantom (sagittal view). PCs are represented as a function of time; their corresponding weights are visualized as a 2D map. The numbers above the PCs indicate the percentage of total variance explained by the corresponding PC.

**Figure 7:**
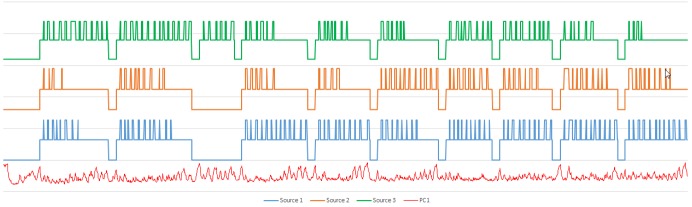
Signal of phantom with source activity. Cobalt-60 sources, co-registered in time with PC1 from daily quality assurance (QA) phantom. The changes in the Cobalt-60 activity correspond to: OFF – Bottom state, READY – Middle state, ON – Upper state. Each of the three sources is offset for legibility.

**Figure 8:**
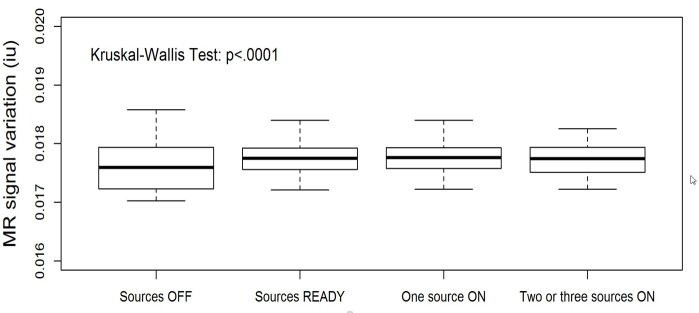
Statistical analysis of third fraction phantom. Box plots of signal intensities in PC1 from principal component analysis (PCA) of daily quality assurance (QA) cine as a function of Cobalt-60 states. The signal variation decreases significantly as more sources are in operation (Kruskal-Wallis test, p < 0.0001).

To confirm these observations, we applied the plan for the third fraction to a daily quality assurance (QA) imaging phantom. The results of PCA analysis for this dataset are shown in Figure 6. Note that the upward trend displayed in the first PC in Figure 2 is present again here to a lesser degree. We plotted this data as described above in a time series to create Figure 7. The overall variance of this series was lower than the patient-sourced dataset, which was expected due to the homogeneity of the phantom and lack of confounding factors such as blood-pulsatile motion and patient bulk motion. As before, the signal peaks seen when the majority of the Cobalt-60 sources were in the OFF position are exhibited in this figure. This was verified by statistical analysis of the resultant data (Figure 8). In this result, the difference between all sources OFF and ON was less drastic, but still visible. The results were determined to be statistically significant by the Kruskal-Wallis test, and coincides with a lesser overall variance of the phantom dataset.

## Discussion

The use of MRI in the discovery and treatment of prostate cancer has been long known by the radiological community [6-8]. However, with the advent of different forms of imaging such as mpMRI and diffusion-weighted imaging, the actual implementation of MRI has not changed dramatically since its induction. The use of pre-treatment and post-treatment MRI is only able to capture the discrete state of the tumor at the precise time those exams were taken, requiring repeat sessions to truly understand the effect of treatment. With the particular features of the MRIdian imaging platform, this study was intended to find features or phenomena in the treatment MRI that could assist in visualization of the signal changes during RT.

In this case report, we characterized the changes of MR signal during a single fraction treatment on MRIdian. We found several distortion patterns in the data presumably resulting from the perturbation of the magnetic field due to the moving metal parts in the platform while treatment was being administered. Should these artifactual variations be isolated or otherwise compensated for, it could bring us closer to the ability to visualize the effects of treatment in real time and predict patient outcomes with a greater accuracy than what is currently standard [9].

While the identified variations were not related to physiological processes, our findings confirm the sensitivity of the developed approach to identifying very small fluctuations. Relating these variations to the physical changes that occur during treatment shows the methodical ability of the technique to uncover their underlying sources.

This report has several limitations. Besides the limited number of studies typical for case reports, the main drawback was that the area of the tumor (GTV) was excluded from the analysis. This was because the tracking and boundary are required for RT and appear on the cine. These contours produce movement and thereby the contours have to be excluded in the analysis. A potential remedy for this issue could be drawing the contours at the surface of the skull and thus excluding them from the Field of View of the analyzed area.

The future application of this approach to LINAC-based MRIdian cines, where the instrumental-induced variations are significantly reduced, has the promise to identify patterns that will provide better insight into the underlying physiological changes.

## Conclusions

In this case report, we characterized the changes of MR signals during a single fraction treatment on MRIdian. We found several distortion patterns in the data likely resulting from the perturbation of the magnetic field due to the moving metal parts in the platform while treatment was being administered. While the identified variations were not related to physiological processes, our findings confirm the sensitivity of the developed approach to identify very small fluctuations.
